# The Th17/Treg Ratio, IL-1RA and sCD14 Levels in Primary HIV Infection Predict the T-cell Activation Set Point in the Absence of Systemic Microbial Translocation

**DOI:** 10.1371/journal.ppat.1003453

**Published:** 2013-06-20

**Authors:** Mathieu F. Chevalier, Gaël Petitjean, Catherine Dunyach-Rémy, Céline Didier, Pierre-Marie Girard, Maria Elena Manea, Pauline Campa, Laurence Meyer, Christine Rouzioux, Jean-Philippe Lavigne, Françoise Barré-Sinoussi, Daniel Scott-Algara, Laurence Weiss

**Affiliations:** 1 Institut Pasteur, Régulation des infections rétrovirales, Paris, France; 2 Université Paris Diderot, Sorbonne Paris Cité, Paris, France; 3 INSERM U1047, Université Montpellier 1, UFR de Médecine, Nîmes, France; 4 Laboratoire de Bactériologie, CHU Carémeau, Nîmes, France; 5 AP-HP, Hôpital Saint-Antoine, Paris, France; 6 AP-HP, Hôpital Européen Georges Pompidou, Paris, France; 7 INSERM U 1018, AP-HP, Université Paris Sud, Paris, France; 8 AP-HP, Hôpital Necker-Enfants Malades, Laboratoire de Virologie, Paris, France; 9 Université Paris Descartes, Sorbonne Paris Cité, Paris, France; Emory University, United States of America

## Abstract

Impairment of the intestinal barrier and subsequent microbial translocation (MT) may be involved in chronic immune activation, which plays a central role in HIV pathogenesis. Th17 cells are critical to prevent MT. The aim of the study was to investigate, in patients with primary HIV infection (PHI), the early relationship between the Th17/Treg ratio, monocyte activation and MT and their impact on the T-cell activation set point, which is known to predict disease progression. 27 patients with early PHI were included in a prospective longitudinal study and followed-up for 6 months. At baseline, the Th17/Treg ratio strongly negatively correlated with the proportion of activated CD8 T cells expressing CD38/HLA-DR or Ki-67. Also, the Th17/Treg ratio was negatively related to viral load and plasma levels of sCD14 and IL-1RA, two markers of monocyte activation. In untreated patients, the Th17/Treg ratio at baseline negatively correlated with CD8 T-cell activation at month 6 defining the T-cell activation set point (% HLA-DR^+^CD38^+^ and %Ki-67^+^). Soluble CD14 and IL-1RA plasma levels also predicted the T-cell activation set point. Levels of I-FABP, a marker of mucosal damages, were similar to healthy controls at baseline but increased at month 6. No decrease in anti-endotoxin core antibody (EndoCAb) and no peptidoglycan were detected during PHI. In addition, 16S rDNA was only detected at low levels in 2 out 27 patients at baseline and in one additional patient at M6. Altogether, data support the hypothesis that T-cell and monocyte activation in PHI are not primarily driven by systemic MT but rather by viral replication. Moreover, the “innate immune set point” defined by the early levels of sCD14 and IL-1RA might be powerful early surrogate markers for disease progression and should be considered for use in clinical practice.

## Author Summary

Generalized immune activation is pivotal in the pathogenesis of HIV disease. Impairment in the gut mucosal barrier allows the translocation of microbial flora from the gut towards the circulation. Translocated microbial products, together with HIV replication, contribute to chronic immune activation. Th17 cells are involved in epithelial barrier integrity and a loss of the balance between Th17 and regulatory T cells (Tregs) has been associated with disease progression. Early events occurring following infection are crucial for the subsequent disease progression. Thus, a high immune activation set point (level of T-cell activation established at the end of acute infection) is a marker of poor prognosis. Whether microbial translocation contributes to the immune activation set point remains an outstanding question. In our longitudinal prospective study of patients with acute infection, we investigated the early relationships between the Th17/Treg balance, monocyte activation and microbial translocation and their impact on the T-cell activation set point. We demonstrated that systemic microbial translocation does not occur at the time of acute infection. Moreover, we identified IL-1RA as a novel plasma biomarker predictive of the immune activation set point. This biomarker could be considered for use in clinical practice as a surrogate marker for disease progression.

## Introduction

High levels of immune activation occur early in primary HIV infection (PHI) and the CD8 T-cell activation set point (i.e. the steady state level of activation following PHI) is a strong predictor of subsequent CD4 T-cell loss independently of viral load [1]. Generalized immune activation is known to be a major contributor to HIV-1 pathogenesis [2]. Although immune activation is dramatically reduced by antiretroviral treatment, residual immune activation remains in virally suppressed ART-treated patients and is associated with poor immune reconstitution [3] and increased morbidity/mortality in treated patients [4].

Impairment of the intestinal barrier and subsequent microbial translocation might be one of the main causes of chronic T-cell activation [5], together with innate and adaptive immune responses, stimulation by HIV viral proteins and reactivation of other viruses (e.g. cytomegalovirus, hepatitis viruses) (reviewed in Appay and Sauce [6]). Microbial translocation leads to the release of bacterial products such as lipopolysaccharide (LPS) which induce monocyte activation, as demonstrated *in vitro* and in different clinical situations including sepsis [7], [8]. LPS levels were shown to be elevated in chronic HIV infection – but not significantly during PHI – and to correlate with T-cell activation [9]. In viremic chronic HIV-infected patients, the spontaneous production of IL-1 by circulating monocytes suggested that these cells were activated *in vivo* [10]. To which extent monocyte activation was caused by the virus and/or by microbial translocation remained unclear. More recently, plasma levels of the monocyte activation marker, soluble CD14 (sCD14) were found to correlate with LPS amounts [9], [11]. Furthermore, sCD14 levels were shown to predict mortality in chronically HIV-infected patients [12] as well as in other contexts (e.g. hemodialysis patients) [13]. Whether systemic microbial translocation occurs and causes immune activation during primary HIV infection has been suggested but not clearly demonstrated. How much immune activation is caused by microbial translocation and when does microbial translocation begin during HIV infection remain outstanding questions [14].

Th17 cells might be crucial in the maintenance of the intestinal mucosal barrier integrity and in the control of microbial translocation [15]. These cells were reported to be depleted in advanced HIV or SIV disease, but preserved in patients with slow disease progression, including elite controllers [16]. Indeed, reduced Th17 cell frequency has also been found in patients with high viral load [17] or low CD4 T cell count [18]. Although exerting mostly opposing functions, Th17 and Treg cells are two closely related CD4 T-cell subsets sharing reciprocal maturation pathways [19]. There is an active balance between the development of either Tregs or Th17 cells and even plasticity between the two subsets [20].The imbalance between Th17 and Tregs has been involved in different settings including autoimmune diseases and cancer [21], [22]. In HIV infection, Tregs might expand following immune activation; however, the increase in Treg frequency was mostly reported as inadequate resulting in a failure to dampen high generalized immune activation in viremic patients [23]. A loss in Th17 to Treg balance has been found in pathogenic SIV infection [24]. Moreover, the Th17/Treg ratio was shown to be lower in progressors compared to elite controllers and was reported to be inversely related to systemic T cell activation in rectosigmoid biopsies from chronically infected patients [18].

In order to decipher the respective role of viral replication and microbial translocation on the establishment of the T-cell activation set point, we investigated, in patients with acute HIV infection, the early relationship between the Th17/Treg balance, monocyte activation and systemic microbial translocation and their impact on the T-cell activation set point, known to predict the rate of CD4 T-cell decline.

## Results

### Patients' characteristics

Twenty-seven patients diagnosed early during PHI (median of estimated time post-infection: 42 days) were prospectively enrolled in the study between June 2009 and December 2011. Patients' clinical characteristics at baseline and at month 6 (M6) of follow-up are depicted in Table 1. A subgroup of these patients have been previously described [25]. Thirteen patients remained untreated during the study period. Ten patients were treated with cART just after baseline sampling; two patients were treated between M3 and M6. Two patients were lost to follow-up. T-cell activation levels were determined by the proportion of cells that expressed the CD38, HLA-DR and/or Ki-67 activation markers, as illustrated in Figure S1.

**Table 1:** Patients' characteristics.

Time points	Patients	HIV-1 RNA *(log_10_/mL)*	CD4 *(%)*	CD4 count *(cells/mm^3^)*	CD8 *(%)*	CD8 count *(cells/mm^3^)*	CD4/CD8 ratio
**Baseline**	untreated	5.65	26	490	54	1117	0.48
	n = 27	(4.57–6.25)	(17–34)	(337–615)	(42–64)	(553–1569)	(0.28–0.78)
**M6**	untreated	4.40	34	669	44	713	0.82
	n = 13*	(3.50–4.80)	(29–38)	(457–725)	(39–49)	(600–924)	(0.62–1.02)
**M6**	ART-treated	1.30	45	750	35	614	1.33
	n = 12**	(1.00–1.33)	(35–48)	(593–783)	(28–37)	(533–672)	(0.99–1.66)

Data are expressed as median (IQR).

* For one patient experiencing an intercurrent episode associated with high level of inflammation at M6, clinical data from M3 were used.

** Twelve patients received ART between baseline and M6. Two patients were lost to follow-up.

**Figure 1:**
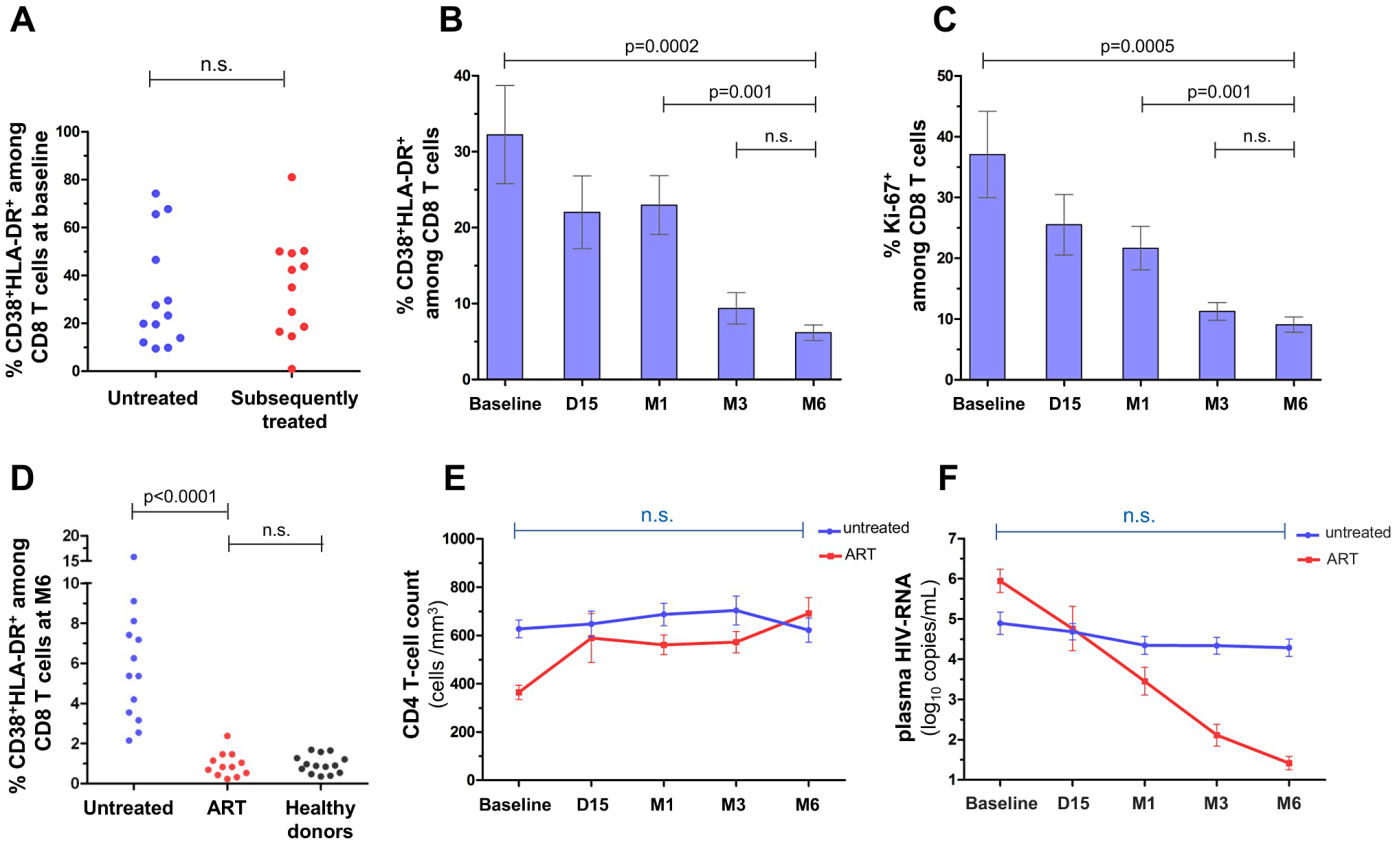
Longitudinal follow-up of CD8 T-cell activation, CD4 cell counts and of plasma HIV-RNA levels in patients with primary HIV infection. At baseline (Panel A), frequencies of CD38^+^HLA-DR^+^ CD8 T cells were compared in patients that remained untreated during the 6-month follow-up (n = 13, Untreated) and in patients that have been subsequently treated before M6 (n = 12, Subsequently treated). CD8 T-cell activation was longitudinally assessed in untreated patients (n = 13) by measuring the frequency of CD38^+^HLA-DR^+^ cells (Panel B) and of Ki-67^+^ cells (Panel C) among CD8^+^ T cells at baseline, day 15 (D15), month 1 (M1), month 3 (M3) and month 6 (M6). Panel D illustrates the proportion of CD38^+^HLA-DR^+^ cells among CD8 T cells in untreated patients, in ART-treated patients at M6 and in healthy controls (n = 14). CD4 T cell counts (Panel E) and plasma HIV-1 RNA levels (Panel F) were plotted as a function of time during the 6 months of follow-up in treated (blue lines) and untreated (red lines) patients. Data are expressed as mean ± SEM. Wilcoxon rank tests were performed and p values are indicated between indicated time points for untreated patients (Panels B, C, E, F). Mann-Whitney tests were performed to compare groups of patients (Panels A and D).

ART-treated and untreated patients did not differ for CD4 and CD8 T-cell activation at baseline (Figure 1A). In untreated patients, the proportion of double positive HLA-DR/CD38 CD8 T cells and of Ki-67-expressing CD8 T cells significantly decreased between baseline and M6 (p = 0.0002 and p = 0.0005 respectively) (Figure 1B and 1C) although T-cell activation levels remained higher than in ART-treated patients (Figure 1D). Interestingly, levels of double positive HLA-DR/CD38 CD8 T cells in those patients, initiating ART early at the time of primary infection were similar to that measured in healthy seronegative controls (Figure 1D). CD8 T-cell activation remained stable between M3 and M6, indicating that the immune activation set point was reached by month 6 of follow-up. CD4 T-cell counts did not change significantly during the 6 months of follow-up (Figure 1E). Median HIV-RNA plasma levels did not significantly decrease during the study period (Figure 1F). A decrease >0.5 log_10_/mL was observed in 5 untreated patients whereas in most untreated patients (8/13), viral set point was reached before inclusion in the study. At baseline, we found a strong positive correlation between plasma viral load and the frequency of CD8 T cells co-expressing CD38 and HLA-DR (r = 0.66, p = 0.0002) or expressing Ki-67 (r = 0.77, p<0.0001) (data not shown).

### Characterization of Th17 cells

**Figure 2:**
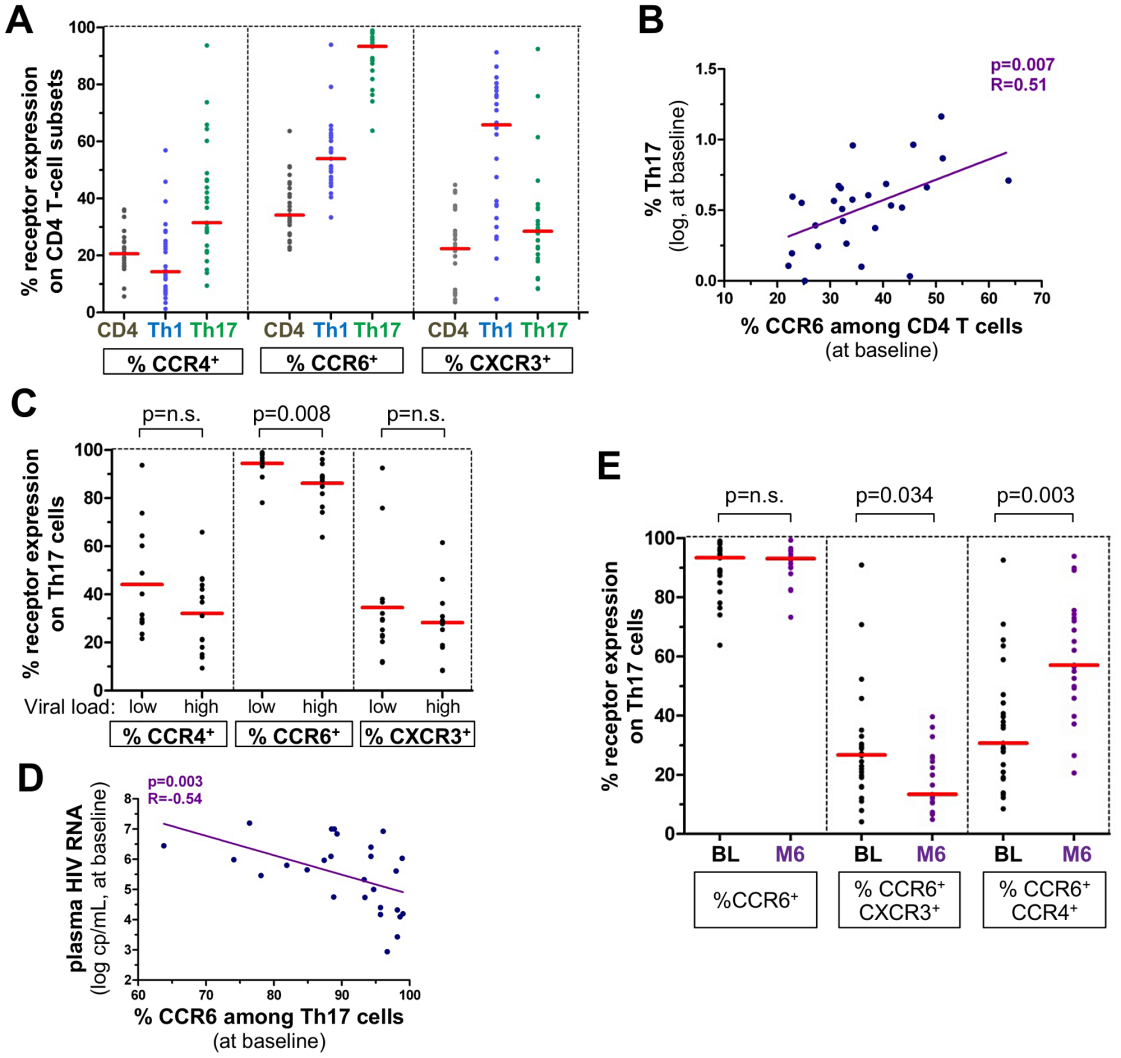
Expression of chemokine receptors by Th17 cells during primary HIV infection. Th1 and Th17 cells were assessed after 5 h PMA/ionomycin stimulation of fresh isolated CD4 T cells and defined by the expression of IFN-γ and IL-2 (Th1) or IL-17 (Th17). In each datasets, horizontal lines indicate the medians. Panels A-D show results at baseline; panel E illustrates both baseline and month 6 measurements. (Panel A) Frequency of CCR4, CCR6 and CXCR3 among bulk CD4 T cells, Th1 cells and Th17 cells. (Panel B) Correlation between the proportion of CCR6 expressing CD4 T cells and the Th17 frequency. Spearman's rank correlation coefficients ‘R’ and corresponding p values are indicated. (Panel C) Expression of CCR4, CCR6 and CXCR3 among Th17 cells in patients with high (>median) and low (<median) viral load at baseline. Median plasma HIV-RNA level was 5.65 log copies/mL. P values according to Mann-Whitney tests are indicated. (Panel D) Correlation between CCR6 expression on Th17 cells and plasma viral load. (Panel E) Expression of CCR6, coexpression of CCR6 and CXCR3 or CCR4 on Th17 cells at baseline and month 6 of follow-up. Wilcoxon rank tests were performed and p values are indicated.

At baseline, a median (IQR) of 3.7% (2.4–4.7) of isolated CD4+ T cells produced IL-17 following stimulation with a combination of PMA and ionomycine. At month 6, there was a trend to a decrease in Th17 cell frequencies (median (IQR) of 2.38% (1.9–3.6) when considering all patients treated or untreated (p = 0.08), the two groups showing similar Th17 levels at M6 (Figure S2)). As illustrated in Figure 2A, most Th17 cells expressed the chemokine receptor CCR6. A median of 31.5% of Th17 cells expressed CCR4. In contrast, CXCR3 was less frequently expressed on Th17 cells compared to IL-2 and IFN-γ-producing Th1 cells (median: 28.5% vs 65.8%). The proportion of CCR6-expressing T cells was directly correlated to the frequency of Th17 cells (r = 0.50, p = 0.007) (Figure 2B). As CCR6^+^ CD4 T cells were reported to be highly permissive to HIV infection [26], we analyzed the impact of HIV replication *in vivo* on the expression of CCR6 by Th17 cells. Less Th17 cells expressed CCR6 in patients with high viral load (i.e. above median of 5.65 log copies/mL) compared to patients with low viral load (i.e. below median) (p = 0.008) whereas Th17 cells expressed similar levels of CCR4 and CXCR3 in both groups (Figure 2C). Accordingly, CCR6 expression on Th17 cells was negatively correlated to plasma HIV-RNA levels (r = −0.54, p = 0.003) (Figure 2D). The expression of CCR6 on Th17 cells was unchanged between baseline and month 6. However, CCR6/CXCR3 co-expression on Th17 cells decreased at month 6 (p = 0.03) while CCR6/CCR4 co-expression increased (p = 0.003) (Figure 2E).

### The Th17 cell frequency and Th17/Treg ratio negatively correlate with viral load and T-cell activation at baseline

We put forward the hypothesis that Th17 cells and/or the balance between Th17 and Tregs could impact the level of immune activation in early PHI. Therefore, we investigated the relationship between *ex vivo* CD4 and CD8 T-cell activation levels and the proportion of IL-17-expressing cells. Tregs were defined as CD4^+^CD25^+^CD127^low^FoxP3^+^ T cells.

**Figure 3:**
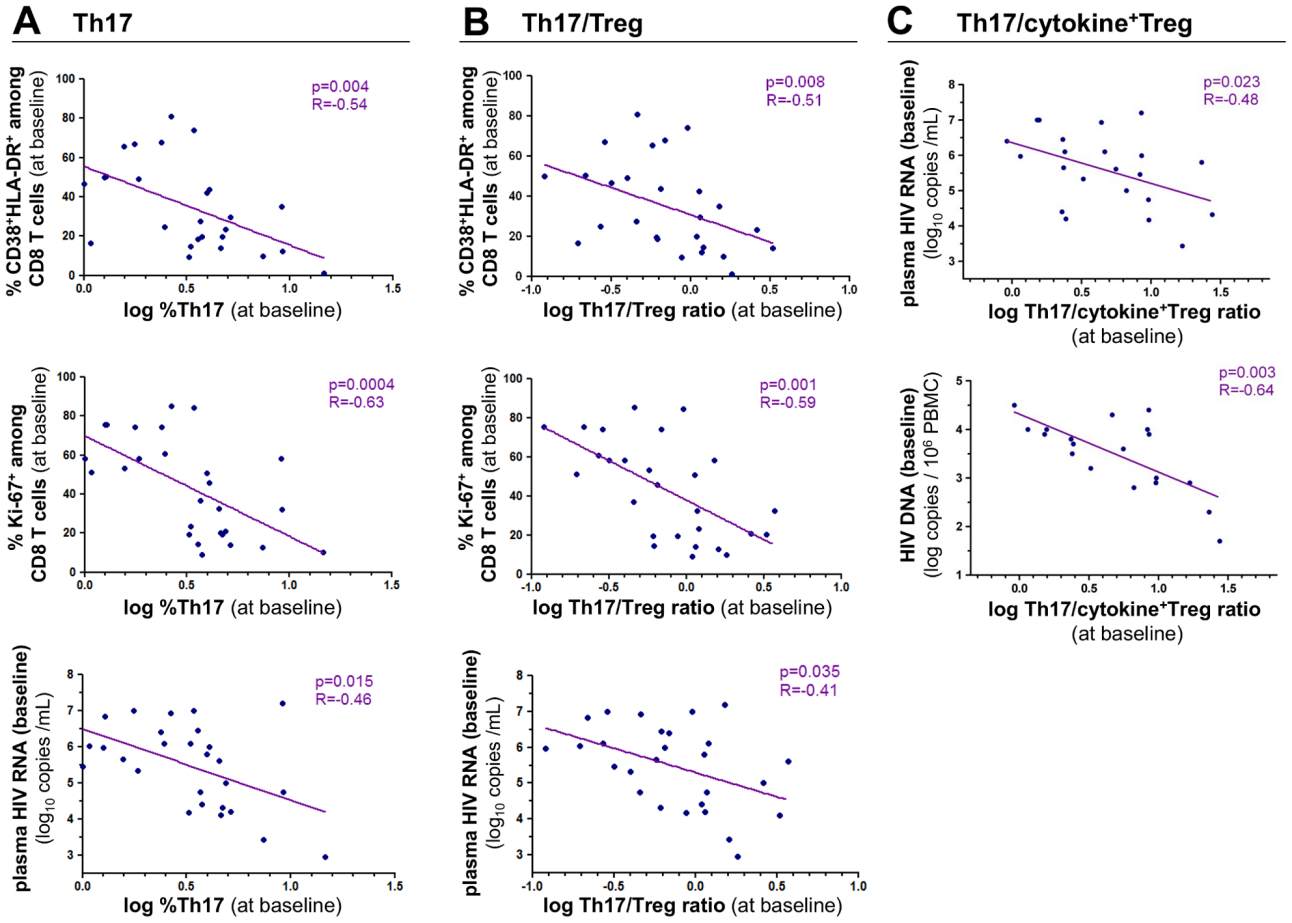
Th17 cell frequency and Th17/Treg ratio negatively correlate with CD8 T-cell activation level and viral load at baseline. Th17 cell frequency was assessed as described in legend to Figure 2. The frequency of Treg cells was defined as CD4^+^CD25^+^CD127^low^FoxP3^+^ cells among CD4 T cells *ex-vivo*; cytokine secreting Tregs were assessed following 5 hrs PMA/ionomycin stimulation of isolated CD4^+^CD25^+^ T-cells and defined as FoxP3^+^ cells expressing IL-10 and/or TGF-β. CD8 T-cell activation was defined by the percentage of CD8 T cells coexpressing CD38 and HLA-DR or CD8 T cells expressing Ki-67. Viral load was expressed as plasma HIV-1 RNA log_10_ copies/mL or HIV-1 DNA log copies/10^6^ PBMCs. The Th17 frequency (Panel A), and the Th17/Treg ratio (Panel B) were illustrated as a function of T-cell activation and viral load in PHI patients (n = 27) at baseline. The Th17/cytokine^+^ Treg ratio (Panel C) was plotted as a function of plasma and cell-associated viral load. Spearman's rank correlation coefficients ‘R’ and corresponding p values are indicated on each panel.

We found a strong negative relationship between the proportion of Th17 cells and the level of CD8 T cells that co-expressed CD38 and HLA-DR at baseline (r = −0.54, p = 0.004) as well as with the proportion of Ki-67-expressing CD8 T cells (r = −0.63, p = 0.0004) (Figure 3A). CD4 T cell activation as measured by HLA-DR expression on CD4 T cells was also negatively correlated with the percentage of Th17 cells (r = −0.52, p = 0.006) (data not shown). In addition, the proportion of Th17 cells negatively correlated at baseline with HIV-RNA plasma levels (r = −0.46, p = 0.015) (Figure 3A) and with HIV-DNA in PBMCs (r = −0.49, p = 0.021) (data not shown). We previously reported that Treg cell frequency was not correlated with CD4 or CD8 T-cell activation [25]. Considering the loss in Th17 to Treg balance reported in pathogenic SIV infection [24], we assessed the relationship between CD8 T-cell activation and the Th17/Treg ratio and found results similar to those observed with Th17 levels (Figure 3B). Of note, Th1 responses as defined by IL-2 or IFN-γ expressing CD4 T cells did not correlate with immune activation or plasma viral load (data not shown). We also assessed Tregs by measuring TGF-β and/or IL-10-producing Foxp3^+^ cells among isolated CD4^+^CD25^+^ T cells following PMA/Ionomycin stimulation. The Th17 to cytokine-expressing Treg ratio also negatively correlated with both HIV-RNA plasma levels and HIV-DNA levels in PBMCs (Figure 3C).

### The Th17/Treg balance is related to monocyte activation at baseline

**Figure 4:**
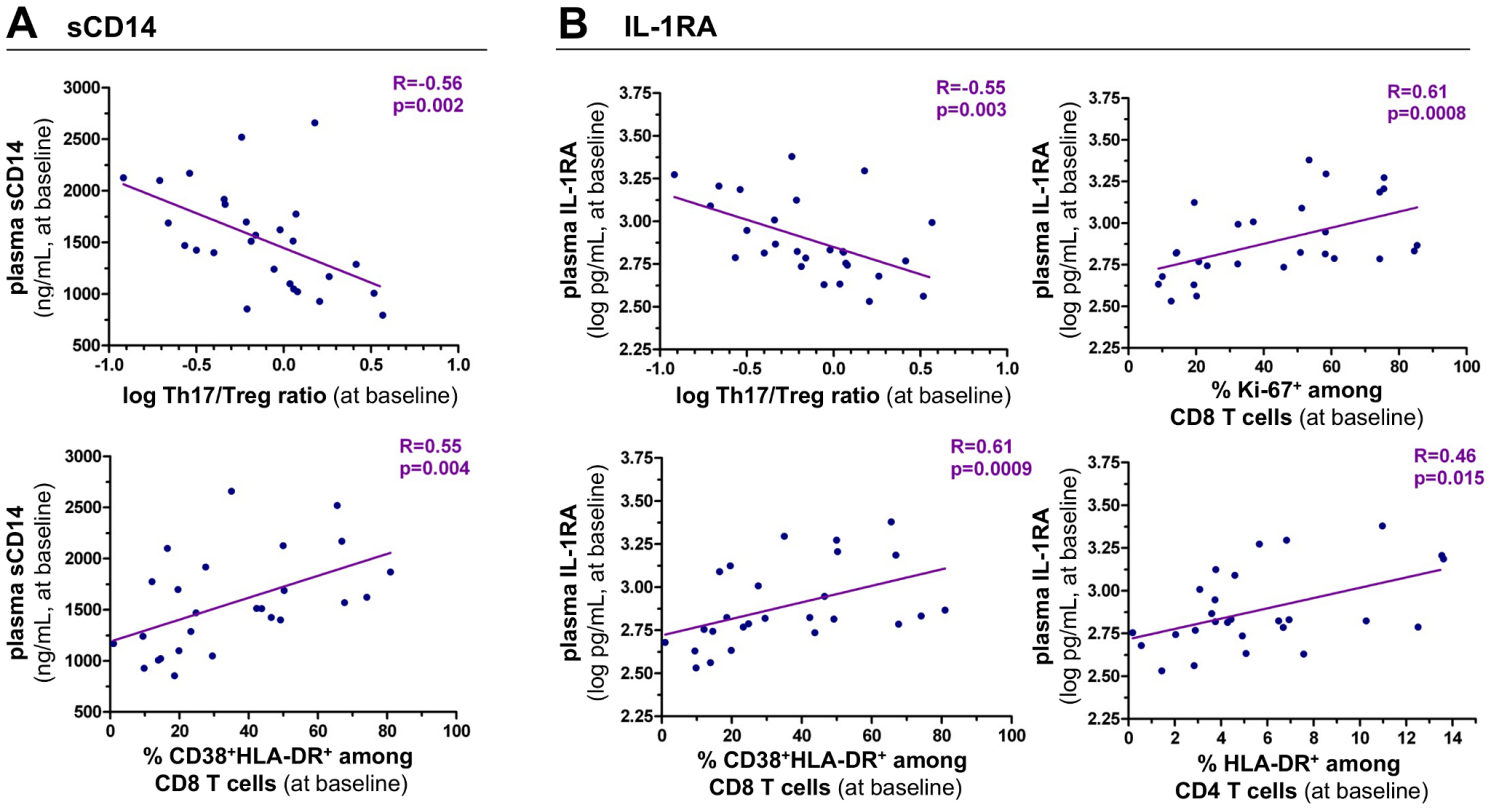
sCD14 and IL-1RA are associated with T-cell activation and negatively correlate with the Th17/Treg ratio at baseline. Baseline plasma levels of sCD14 (Panel A) and IL-1RA (Panel B) were plotted as a function of the Th17/Treg ratio and of T-cell activation in study patients (n = 27). CD8 T-cell activation was defined by the proportion of CD8 T cells coexpressing CD38 and HLA-DR or expressing Ki-67. CD4 T-cell activation was defined by the percentage of CD4 T cells expressing HLA-DR. Spearman's rank correlation coefficients ‘R’ and corresponding p values are indicated on each panel.

Lower Th17 cells being associated with higher T-cell activation, one may hypothesize that T-cell activation results from microbial translocation through monocyte activation. Thus, we focused on the relationship between the Th17/Treg balance and soluble markers of monocyte activation. Plasma levels of sCD14 and IL-1RA did not significantly change between baseline and M6 and were similar in untreated and treated patients (Figure S3). At baseline, sCD14 plasma levels negatively correlated with the Th17/Treg ratio (r = −0.56, p = 0.002), and was positively related to CD8 T-cell activation (r = 0.55, p = 0.004) (Figure 4A) but not to CD4 T-cell activation, as assessed by the proportion of HLA-DR expressing CD4 T cells (data not shown). In addition to sCD14, which binds LPS but is also an acute phase protein, we investigated IL-1RA as a cytokine antagonist secreted by activated monocytes in concert with the pro-inflammatory cytokine IL-1 [27]. We found a strong negative relationship between plasma levels of IL-1RA and the Th17/Treg ratio (r = −0.55, p = 0.003). IL-1RA was positively associated with CD38^+^HLA-DR^+^ (r = 0.61, p = 0.0009) and Ki-67^+^ CD8 T cells (r = 0.61, p = 0.0008). IL-1RA was also found to be positively related to HLA-DR^+^ CD4 T cells (r = 0.46, p = 0.015) (Figure 4B). Of note, MIP-1α, highly expressed in LPS-stimulated monocytes, was below limit of detection (10 pg/mL) in 24 of the 27 patients.

### Lack of systemic microbial translocation

Considering the lack of direct demonstration of MT in acute HIV infection, we were interested to further investigate evidence of mucosal damage and gauged the levels of microbial translocation in the study population. Fatty acid-binding proteins (FABPs) are plasmatic markers of tissue injuries. Intestinal-FABP (I-FABP) can be detected in plasma after leaking out of damaged enterocytes from the small intestine [28], [29].

**Figure 5:**
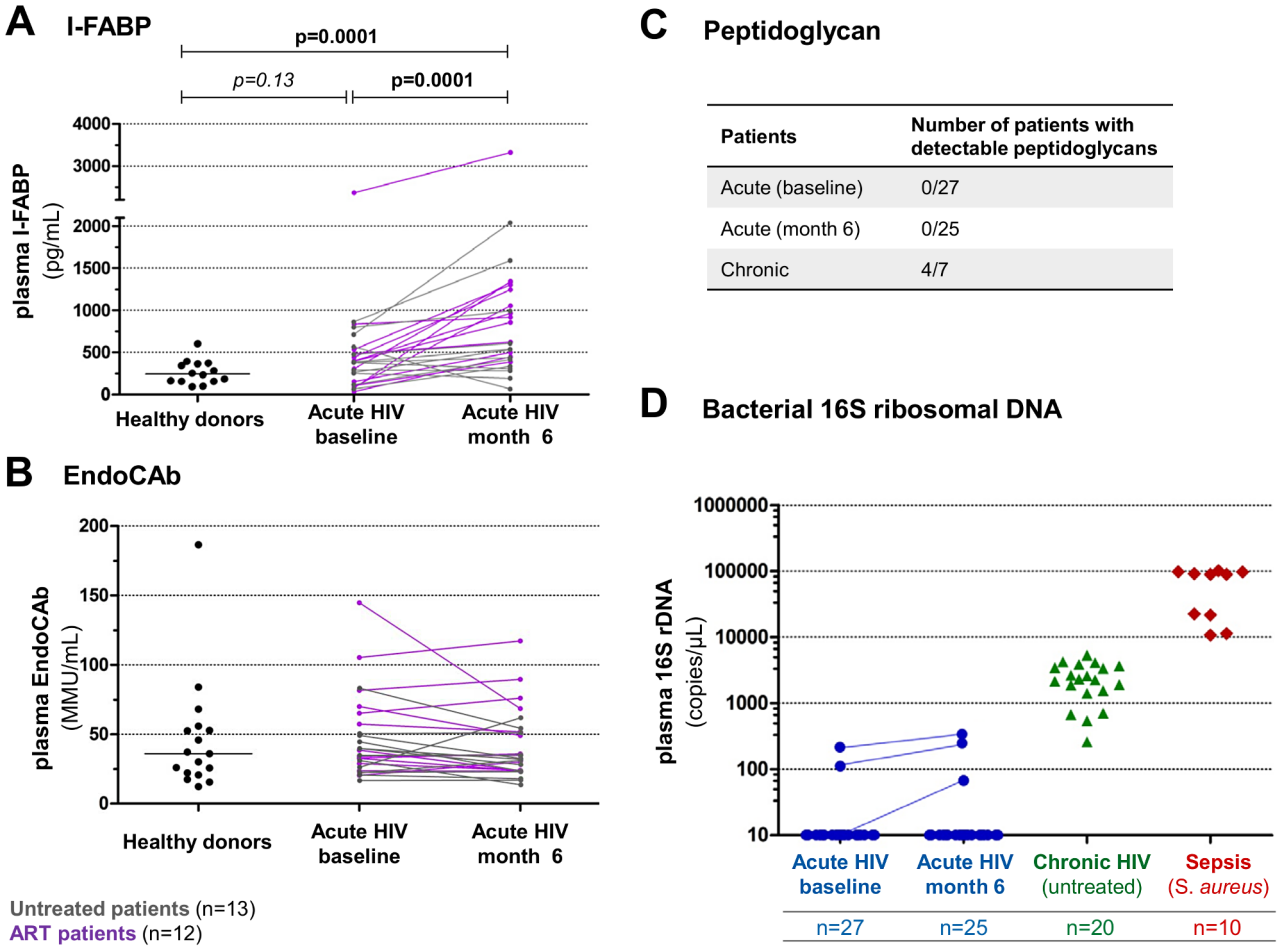
Intestinal mucosal integrity and microbial translocation markers during primary HIV infection. Plasma levels of I-FABP (Panel A) and plasma EndoCAb IgM levels (Panel B) were measured in PHI patients at baseline and month 6 and in healthy donors. Purple lines indicate patients receiving antiretroviral therapy between baseline and month 6; grey lines indicate untreated patients. Mann-Whitney tests were performed to compare patients and healthy donors, Wilcoxon rank tests was used to compared baseline and month 6 values in all patients; significant p values are indicated. (Panel C) Number of patients with detectable peptidoglycans in plasma using SLP reagents. (Panel D) Plasma levels of 16S ribosomal DNA in PHI patients at baseline (n = 27) and month 6 (n = 25), in untreated chronically HIV-infected patients (n = 20) as well as in patients with sepsis and blood culture positive for *Staphylococcus aureus* (n = 10).

At baseline, levels of I-FABP were similar in patients and healthy donors. However, we found a significant increase in I-FABP levels from baseline to M6 (p = 0.0001 when considering all treated and untreated patients) (Figure 5A). Of note, I-FABP also increased in treated patients at month 6 (p = 0.0005). No relationship was found between Th17 frequency and I-FABP levels (data not shown).

The hypothesis of the occurrence of MT in patients with acute/early HIV infection was suggested by a decrease in anti-LPS antibodies (EndoCAb), rather than the presence of LPS in plasma [9]. In the present study involving patients with early primary HIV infection, baseline EndoCAb levels did not differ from those of healthy donors (Figure 5B). In addition, EndoCAb levels did not change between baseline and M6 and did not correlate with T-cell activation (Figure 5B and data not shown).

Apart from LPS, peptidoglycan (PGN), another microbial component, could be found in plasma following microbial translocation. In contrast to patients with chronic infection, in whom we detected plasma pepditoglycan (in 4 of 7 tested patients), we found no detectable peptidoglycan in any of the 27 PHI patients (Figure 5C). To avoid misinterpretation due to the SLP test sensitivity, we also quantified the 16S ribosomal DNA by quantitative PCR in all plasma samples at baseline and M6. Bacterial rDNA was only detected in 2 out of 27 patients at baseline and in one additional patient at M6. Moreover, plasma levels of 16S rDNA in these three patients were low (<400 copies/µL at month 6) compared to the levels detected in 20 patients with chronic untreated HIV infection (median: 2.285 copies/µL) and in 10 patients with sepsis and blood cultures positive for *Staphylococcus aureus* (median: 89.100 copies/µL) (Figure 5D).

### The Th17/Treg balance and IL-1RA at baseline predict the T-cell activation set point

**Figure 6:**
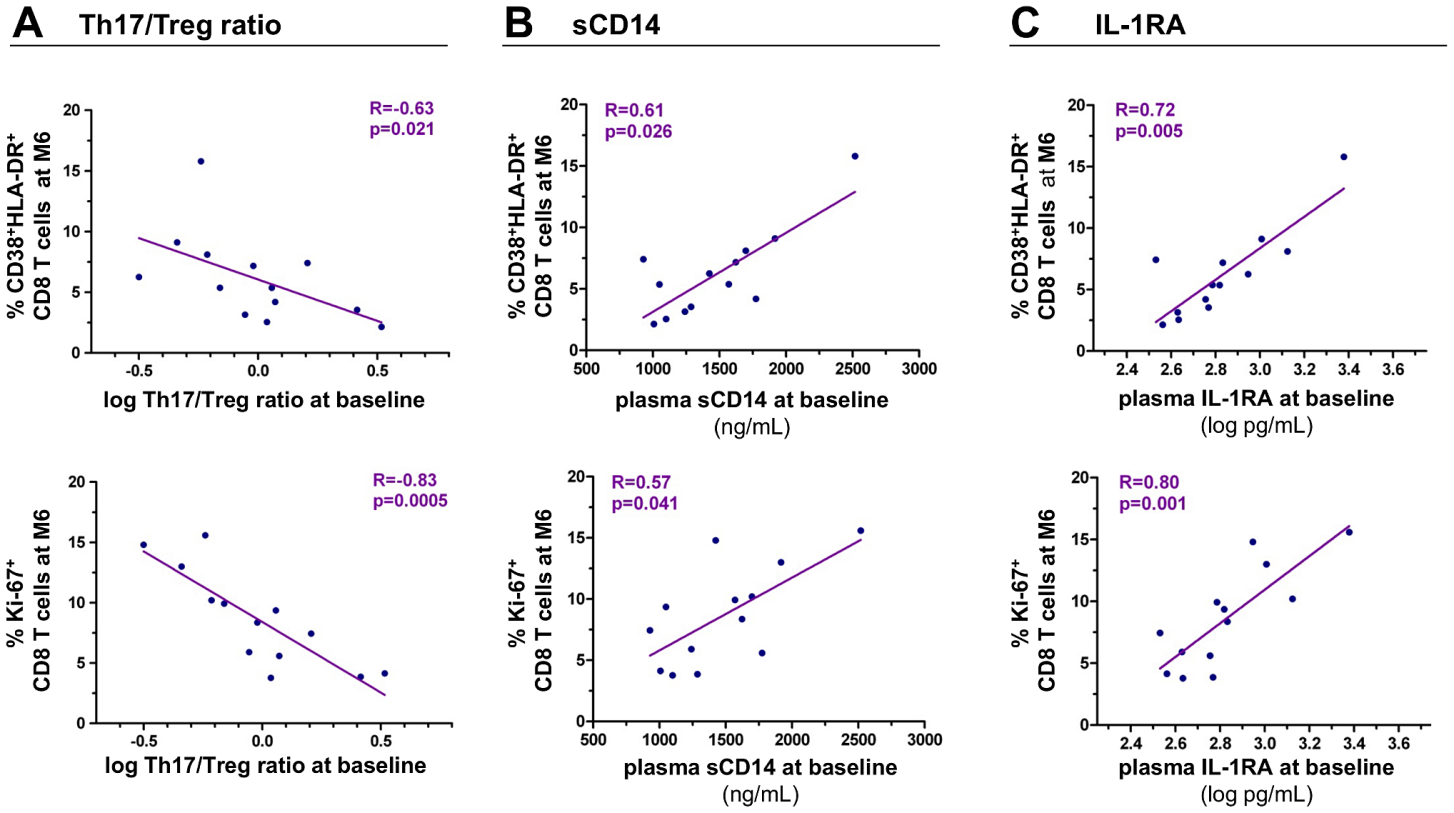
The Th17/Treg ratio and monocyte activation markers at baseline predict the T-cell activation set point. Panels depict the relationships between the Th17/Treg ratio (Panel A), plasma levels of sCD14 (Panel B) or IL-1RA (C) at baseline and CD8 T cell activation at month 6. The T-cell activation set point was defined as the frequency of CD8 T cells co-expressing CD38 and HLA-DR or expressing Ki-67 at month 6. Spearman's rank correlation coefficients ‘R’ and corresponding p values are indicated on each panel.

Finally, we assessed the impact of the Th17 to Treg balance at baseline on the T-cell activation set point after 6 months of follow-up in untreated patients. The Th17/Treg ratio at baseline negatively correlated with the proportion of CD38^+^HLA-DR^+^ CD8 T cells at month 6 (r = −0.63, p = 0.020) as well as with Ki-67-expressing CD8 T cells (r = −0.83, p = 0.0005) (Figure 6A).

We also investigated whether soluble markers of monocyte activation, shown to correlate with the Th17/Treg ratio, can also predict the T-cell activation set point. Levels of sCD14 at baseline correlated with the proportion of CD8 T-cells co-expressing CD38 and HLA-DR at month 6 (r = 0.61, p = 0.026) and with Ki-67 expression in CD8 T-cells (r = 0.57, p = 0.041) (Figure 6B). Strikingly, baseline levels of IL-1RA were strongly correlated with CD38^+^HLA-DR^+^ CD8 T cells at month 6 (r = 0.72, p = 0.005) as well as with Ki-67^+^ CD8 T cells (r = 0.80, p = 0.001) (Figure 6C).

## Discussion

The present study shows that the Th17/Treg ratio strongly correlates with the level of generalized T-cell activation in acute HIV infection. The loss of Th17 to Treg balance was found to be associated with elevated plasma levels of monocyte/macrophage activation soluble markers. Here, we show for the first time that IL-1RA plasma levels are associated with T-cell activation and that the early level of IL-1RA is a strong predictor of the T-cell activation set point. In addition, data indicate that, in acute HIV infection, immune activation is closely dependent on viral replication and not on systemic microbial translocation that occurs later in the natural history of infection.

Th17 cells, involved in the maintenance of the intestinal mucosal barrier integrity and in the defence against microbial infections were reported to be depleted in advanced HIV disease [15], [17]. Few data are available regarding Th17 cells in primary HIV infection. Th17 cells were shown to decrease early following SIV infection of pigtail macaques [24]. In the present study, we enrolled patients within approximately 40 days following estimated time of infection. The frequency of Th17 cells did not significantly change between baseline and month 6. In humans, TH17 cells express the chemokine receptors CCR6 and CCR4 whereas Th1 cells mainly express CXCR3 [30]. We found that the great majority of IL-17^+^ cells expressed CCR6. The expression of CCR4 and CXCR3 was heterogeneous among patients; cells that coexpressed CXCR3 and CCR6 were preferentially Th1/Th17 cells, which also produced IFN-γ (data not shown). CCR6^+^ Th17 cells were demonstrated to be highly permissive to HIV infection and preferential targets for the virus [26], [31]. We observed a slight but significant decrease in CCR6 expression on Th17 cells in the group of patients with high viral loads and a negative relationship between the proportion of Th17 cells that express CCR6 and the plasma HIV-RNA levels. These data could result from an early loss of CCR6^+^Th17 cells. Alternatively, activated cells could have downregulated CCR6 expression [32]. From baseline to month 6, the proportion of CCR6^+^ Th17 cells did not change whereas Th17 cells co-expressing CCR6 and CXCR3 decreased while CCR6^+^/CCR4^+^ Th17 cells increased. CCR6^+^CXCR3^+^ cells were reported to express more frequently the HIV co-receptor CCR5 and the gut-homing integrin β7 compared to CCR6^+^CCR4^+^ cells [26]. Thus, a relative depletion of peripheral CCR6^+^CXCR3^+^ IL-17-secreting cells might be related to a preferential targeting of these cells, as well as their migration to the gut.

By preventing microbial translocation, Th17 cells and/or the balance between Th17 and Tregs could impact the level of immune activation in early PHI, as suggested in pathogenic SIV infection [24].

Here, we show that, as in the macaque model, the Th17/Treg ratio at baseline negatively correlated with T-cell activation. In addition this ratio was also inversely related to plasma viral load. It could be postulated that a high Th17/Treg ratio might be associated with the control of microbial translocation. Thus, we measured sCD14 plasma levels as well as other monocyte activation markers including IL-1RA and MIP-1α. We investigated IL-1RA rather than IL-1, that cannot be reliably measured in plasma due to a short half-life and/or to a rapid clearance from circulation [27], [33]. The Th17/Treg ratio was negatively associated with the monocyte activation markers sCD14 and IL-1RA; MIP-1α was undetectable in most patients.

Soluble CD14 is frequently measured as a surrogate marker of microbial translocation but increased levels of sCD14 reflect monocyte activation, whatever the stimulus. We thus assessed markers of mucosal integrity (I-FABP) and of microbial translocation including anti-LPS antibodies (EndoCAB), peptidoglycan and 16S rDNA plasma levels. I-FABP levels have been reported to be higher in HIV-infected ART-treated patients as compared with healthy donors; I-FABP was also found to be associated with lower CD4 T cell counts [12], [34]. In this study, I-FABP levels were increased at month 6 but not significantly at baseline, although statistical significance might have been reached with higher number of patients. The increase in I-FABP levels suggests that the loss of mucosal integrity appeared between baseline and month 6 in most patients. This is consistent with the decreased expression of genes involved in the regulation of epithelial barrier maintenance reported after 1–2 months in primary HIV infection [35]. However, we cannot exclude that impairment of the mucosal barrier occurred earlier before the detection of released I-FABP. Interestingly, early ART initiation did not prevent damages in the intestinal mucosa at month 6 as I-FABP also increased in patients receiving antiretroviral treatment during the study period. There was no correlation between the proportion of IL-17-secreting cells and I-FABP levels. However, the cytokine, produced by Th17 cells, crucial for the maintenance of normal barrier homeostasis and the prevention of dissemination of commensal bacteria is probably IL-22, rather than IL-17 [36], [37]. Accordingly, loss of IL-22^+^ lymphocytes was reported to be associated with mucosal damage in SIV infection [38].

The hypothesis of the occurrence of systemic microbial translocation in patients with acute/early HIV infection was indirectly suggested by a decrease in EndoCAB, since the increase in plasma LPS has been observed in patients with chronic infection but not with acute or “early chronic” infection [9]. It was suggested that LPS could not be detected in acute HIV infection because of naturally occurring EndoCAbs that bind to and clear translocated LPS from the circulation. In this study, EndoCAb levels were similar in patients and in healthy controls and remained unchanged at month 6. In our study, patients were included in the early phase of acute HIV infection which could explain the discrepancy. Of note, it was shown that EndoCAb levels remained stable during HIV disease progression in Africa and that LPS and EndoCAb levels were not correlated [39].

To directly assess the presence of microbial products, we measured plasma levels of peptidoglycan, a major cell-wall component of both Gram-negative and Gram-positive bacteria as well as bacterial 16S rDNA. Unlike chronically infected patients, all plasma samples from patients with acute HIV infection were negative for peptidoglycans. Similar to the Limulus amebocyte lysate assay which was suspected to give inconsistent results and to underestimate microbial translocation in HIV/SIV infection [40], the test used to detect peptidoglycans could fail to detect low levels of this microbial product in plasma samples. We thus also quantified bacterial 16S rDNA by a sensitive quantitative PCR method to detect conserved regions of bacterial DNA. We found no 16S rDNA in most patients with primary HIV infection, in contrast to patients with chronic HIV infection and with sepsis.

Taken together, data indicate the lack of systemic microbial translocation in early PHI, although microbial products may be increased in the gut or liver and cleared before reaching peripheral circulation. Detection of variable levels of sCD14 in the absence of microbial translocation is consistent with previous studies suggesting that sCD14 might be independent of LPS levels, at least in some patients [39], [41]. Our results strengthen the idea that sCD14 and LPS should not be indistinctly used to evaluate microbial translocation in HIV-infected patients.

**Figure 7:**
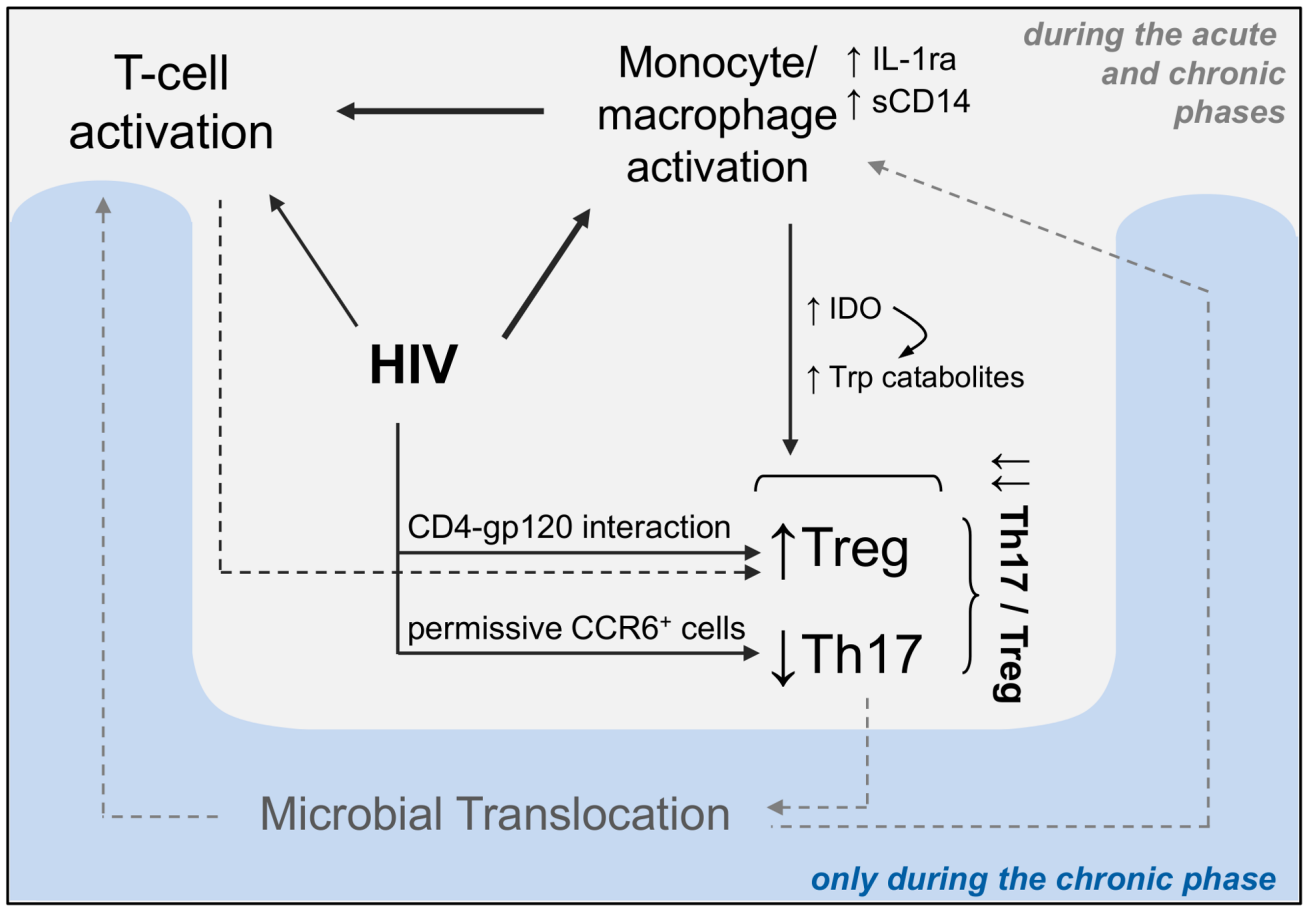
Proposed model of interrelationships between HIV, immune activation and the Th17/Treg ratio. HIV induces monocyte and T-cell activation either directly (e.g. through TLRs) or indirectly, through innate and adaptive responses. The Th17 to Treg ratio decreases as Tregs expand in HIV infection and Th17 cells are one of the preferential targets of the virus. HIV can induce IDO expression by antigen-presenting cells which leads to tryptophan (Trp) catabolites accumulation that inhibit Th17 cells and induce Tregs. This further accentuates the decrease in the Th17/Treg ratio. In addition, IL-1RA secreted by activated monocytes could inhibit Th17 cell development. Then, in a later phase of infection – and not in acute infection – the loss of Th17 cells might contribute to microbial translocation that participates in chronic immune activation.

Data from the present study clearly demonstrate that immune activation in acute HIV infection does not result from systemic microbial translocation. The lack of evidence of microbial translocation at the time of acute infection strongly suggests that early immune activation mainly results from viral replication. This hypothesis is further supported by the absence of MIP-1α, a chemokine highly expressed by LPS-stimulated monocytes not correlated with viral replication [42], [43]. HIV-1 drives monocyte/macrophages towards an inflammatory phenotype following infection and/or through gp120/CD4 interaction [44], [45]. Toll-like receptor (TLR)-independent activation may result in an increased responsiveness of macrophages to TLR ligands [46]. HIV-1 single stranded RNA is recognized by the TLR8 on monocyte/macrophages [47]. A recent study showed that monocyte TNF-α responses following TLR8 stimulation were higher in HIV-infected individuals compared to healthy donors. Interestingly, the percentage of TNF-α -producing monocytes following TLR8 stimulation strongly positively correlated with HIV-1 RNA levels both in acute and chronic HIV-1 infection [48]. We showed that the baseline level of CD8 T-cell activation strongly correlated with sCD14 and IL-1RA as well as with the HIV-RNA plasma levels [25]. Generalized CD8 T-cell activation may result from HIV-induced activation of monocytes/macrophages and from other innate immune responses including the strong cytokine storm detected during the peak of viral replication [49]. Moreover, HIV may directly activate T cells, as suggested by the observation that, in HIV-infected patients, a high expression of TLR7 on purified CD8 T cells was associated with the up-regulation of activation markers following TLR7 stimulation [50]. Besides, HIV-specific CD8 T cells may stand for a substantial part of activated CD8 T cells since major HIV-driven oligoclonal expansions of TCR Vβ subsets of CD8 T cells was reported during acute HIV infection [51]. Moreover, in line with a direct role for the virus on T-cell activation, we found that early ART initiation, at the time of acute infection, decreased CD8 T cell activation at levels similar to that of healthy donors. Both CD8 T-cell activation and soluble markers of monocyte activation were found to be negatively associated with the Th17 to Treg balance. In addition, the Th17/Treg ratio itself negatively correlated with viral load including HIV-DNA in PBMCs. As discussed above, Th17 cells are one of the preferential targets of the virus [26], [31], which may account for the negative relationship observed between HIV-RNA plasma levels and peripheral Th17 cell frequency. On the other hand, HIV induces Treg cell expansion, through direct and indirect mechanisms (reviewed in [52]). In the context of primary HIV infection, interferons, and also HIV itself may drive the production of the enzyme indoleamine 2,3-dioxygenase (IDO) and tryptophan (Trp) catabolism by macrophages and dendritic cells [18], [53], [54]. IDO-mediated metabolism leads to induction of Tregs and inhibition of Th17 differentiation through the accumulation of Trp catabolites [18]. Altogether, this might result in decreased Th17/Treg ratio in patients with high viral replication. We can thus hypothesize that the loss of the Th17 to Treg balance is a consequence of viral replication and immune activation in acute HIV infection. In addition, the alteration of the Th17 to Treg balance could result from the high levels of circulating IL-1RA secreted by activated monocytes. This hypothesis is supported by the demonstration that IL-1RA reduces the differentiation of Tregs into Th17 cells both *in vitro* [55] and *in vivo* in a model of IL-1RA-deficient mice [56] and in humans [57]. The loss of Th17 cells could facilitate microbial translocation and subsequent generalized T-cell activation only in the chronic phase of infection [18], [58] (Figure 7).

We put forward the hypothesis that HIV replication might reduce the Th17/Treg ratio directly and through activation of innate immune cells, so that early Th17/Treg ratio and the level of monocyte/macrophage activation may reflect the intensity of host responses and impact the T-cell activation set point, known to predict disease progression [1]. The level of monocyte activation and the Th17/Treg ratio at baseline were found to predict the CD8 T-cell activation set point. T-cell activation was shown to significantly decrease between baseline and month 6, the immunologic set point being reached between 3 and 6 months of follow-up. In contrast, the levels of monocyte activation and the Th17/Treg ratio did not significantly vary during the first 6 months. Also, HIV-RNA levels did not significantly decrease throughout the follow-up indicating that the viral set point was already reached at the time of inclusion in the study in most patients. Altogether, this suggests that monocyte/macrophage activation paralleled viral replication and that both had already decreased at study baseline while the establishment of the T-cell activation set point was delayed beyond 3 months. These different kinetics of viral replication, monocyte and CD8 T-cell activation probably explain why the level of CD8 T-cell activation at baseline did not predict its own set point at month 6 (data not shown). One may postulate that an “innate immune set point” (i.e. the steady state level of monocyte/macrophage activation) precedes and predicts the T-cell activation set point itself predictive of the rate of subsequent CD4 T-cell decline [1]. Of note, IL-1RA and sCD14 levels only decreased in the few patients with the highest levels at baseline (Figure S3).

In conclusion, the early Th17 to Treg balance as well as sCD14 and IL-1RA levels – that may be indicative of an “innate immune set point” – predict the CD8 T-cell activation set point. Altogether, data support the hypothesis that T-cell activation in acute infection is primarily driven by the HIV-induced innate immune responses and not by systemic microbial translocation, which occurs later in HIV disease. Soluble CD14 and IL-1RA can be easily measured and should be considered for use in clinical practice as early surrogate markers for disease progression. This needs to be confirmed in larger prospective cohorts of patients with primary HIV infection.

## Materials and Methods

### Study population

Twenty-seven individuals with acute HIV infection were enrolled in a prospective study (co-inclusion in the CO6-PRIMO ANRS cohort) conducted in four clinical sites in Paris, France. Acute HIV infection was defined by a negative or weakly positive ELISA, and at least one of the following criteria: less than three bands on HIV Western Blot, a positive p24 antigenaemia or detectable plasma HIV-RNA. The estimated date of infection was calculated as 2 weeks before onset of symptoms for patients with symptomatic PHI (26/27) or 4 weeks before the first positive Western Blot. At baseline (day 0 of enrollment), all patients were treatment-naive. Some of the patients started combination antiretroviral treatment (cART) during the follow-up, based on clinical symptoms, CD4 cell counts (e.g. below 500/mm3 according to French recommendations) and the decision of both physicians and patients. Patients who were treated during the study were receiving a combination of nucleoside analogues, a boosted protease inhibitor and raltegravir with or without maraviroc. Written informed consent was provided by study participants according to French ethical laws. The ethical committee of Ile de France II, approved the study. Blood from patients was collected at baseline, day 15, month 1 (M1), month 3 (M3) and month 6 (M6). Plasma samples were also collected from healthy volunteers (n = 17).

### Flow cytometric analysis

Peripheral blood was collected in EDTA-containing tubes. Fresh peripheral blood mononuclear cells (PBMCs) were purified by density gradient centrifugation (Isopaque-Ficoll) within 2–4 hours after blood sampling. Freshly isolated cells were used the same day and plasma samples were frozen for subsequent use.

After washings, cells were stained using multicolor panels and analyzed by flow-cytometry (LSRII cytometer driven by the FACSDiva software, Becton Dickinson) as described previously [25]. The following monoclonal antibodies (mAbs) conjugated to PE Texas Red (ECD), peridinin chlorophyll protein–cyanin 5.5 (PerCP–Cy5.5), Alexa Fluor 488 (AF488), Alexa Fluor 647 (AF647), Alexa Fluor 700 (AF700), allophycocyanin (APC), allophycocyanin–Hilite7 (APC–H7), phycoerythrin–cyanin 7 (PE–Cy7), phycoerythrin–cyanin 5 (PE–Cy5), fluorescein isothiocyanate (FITC), and phycoerythrin (PE) and eFluor 450 (eF450) were used at predetermined optimal concentrations: anti–CD3–ECD (Beckman Coulter); anti–CD4–PerCP–Cy5.5, anti–CD4–APC–H7, anti–CD8–AF488, anti–CD25–APC, anti–HLA-DR–PerCP–Cy5.5, anti–CD38–APC, anti–CCR4–PE–Cy7, anti–CCR6–PE, anti–CXCR3– PE–Cy5, anti–IL-17–AF647, anti–IL-2–FITC and anti–IFN-γ–AF700 (BD Biosciences); anti–CD127–PE–Cy7, anti–IL-10–eF450, anti–FoxP3–APC and anti–FoxP3–AF700 (eBiosciences); anti–TGF-β–PE (IQ Products) and anti–Ki-67–FITC (Dako). FcR Blocking Reagent (Miltenyi Biotec) was used to block unwanted binding of antibodies and increase the staining specificity of cell surface antigens. For intracellular staining of FoxP3, Ki-67, IL-10, TGF-β, IL-2, IFN-γ or IL-17, cells were fixed and permeabilized using the “FoxP3 Staining Buffer Set” (eBioscience) according to the manufacturer's recommendations. Analyses were performed using FlowJo software (TreeStar).

### Measurement of Th17 cells and cytokine-secreting Tregs

CD4^+^ T-cell enrichment was performed prior to Ficoll-Hypaque density gradient centrifugation by incubating the blood with RosetteSep human CD4 T-cell enrichment antibody coktail (Stem Cell Technologies) according to the manufacturer's instructions. Subsequent enrichment of CD25^+^ cells was performed using EasySep human CD25 positive selection cocktail and the cell separator RoboSep (Stem Cell Technologies). Fresh CD4^+^ T cells and the CD25-enriched fraction were stimulated with PMA (5 ng.mL^−1^) and ionomycin (1 µg.mL^−1^) at 37°C for 5 hours. After 2 hours of culture, brefeldin A (5 µg/mL) (Sigma-Aldrich) was added. Intracellular cytokine staining was performed as described above.

### Enzyme-linked immunosorbent assays

Commercially available enzyme-linked immunosorbent assay (ELISA) kits were used according to the manufacturers' recommendations for measuring concentrations of intestinal fatty acid binding protein (I-FABP) (Hycult Biotech), soluble sCD14 (sCD14) (R&D Systems), IL-1 receptor antagonist (IL-1RA) (eBioscience), Endotoxin Core IgM Antibody (EndoCAb) (Hycult Biotech), MIP-1α (Tebu-bio) in plasma samples. For the measurement of sCD14 and EndoCAb, plasma samples were diluted 1∶1000 and 1∶100 (v/v) in the provided assay diluents, respectively. Results were analyzed using a five parameter-logistic (5PL) function for fitting standard curves obtained from recombinant protein standards.

### Peptidoglycan assay

The Silkworm Larvae Plasma (SLP) reagent set (Wako Pure Chemical Industries) was used to quantify peptidoglycans (PGN) in plasma samples [59]. Plasma were diluted at a 1∶10 ratio in sterile water and heated for 10 minutes at 80°C. Samples and an equal volume of reconstituted SLP reagent were mixed in a 96-well plate. The OD_650_ was measured after 1 hr incubation at 30°C. The amount of PGN was calculated using a standard curve obtained with digested PGN from *S. aureus* (Wako Pure Chemical Industries) serially diluted and heated in plasma from healthy donors that were previously tested as PGN free.

### Plasma DNA isolation and 16S ribosomal DNA quantification

DNA was extracted from 200 µL of plasma using the DNeasy Blood and Tissue Kit (Qiagen), according to the manufacturer's instructions. A NanoDrop 2000 spectrophotometer (Thermo Scientific) was used to determine DNA concentrations.

Bacterial 16S rDNA levels were measured by quantitative polymerase chain reaction (PCR). A 20 µL amplification reaction consisted of 2 µL of 10× PCR buffer (100 mmol/L Tris-HCl, pH 8.3; and 500 mmol/L KCl [Invitrogen]), 3.5 mmol/L MgCl2, 0.2 mmol/L deoxynucleoside triphosphate, 0.5 µmol/L forward and reverse primers, 0.75 U of Taq polymerase (Invitrogen), and 5 µL of the template plasma DNA. Degenerate forward (8F: 5′-AGAGTTTGATYMTGGCTCAG) and reverse (361R: 5′-CGYCCATTGBGBAADATTCC) primers were used to amplify DNA templates encoding 16S rRNA. The DNA was amplified in triplicate, and mean values were calculated. A standard curve was created from serial dilutions of plasmid DNA containing known copy numbers of the template. The reaction conditions for amplification of DNA were 94°C for 5 min, followed by 45 cycles at 94°C for 10 s, 54°C for 45 s and at 72°C for 60 s. The assays were performed using a LightCycler 480 (Roche). The experiment was performed twice and positive samples were tested a third time.

### Virological assays

Plasma HIV-RNA levels were determined on site, using the locally available technique with a detection limit of 20 copies/mL. The HIV DNA level in PBMCs was quantified in whole blood using the “Agence Nationale de Recherches sur le Sida et les Hépatites Virales” (ANRS) real-time PCR method (Biocentric, Bandol, France), as previously described [60]. Results were expressed as the log_10_ number of HIV-1 DNA copies per 10^6^ PBMCs (threshold: 60 copies/10^6^ PBMCs).

### Statistical analysis

Data were described by medians and interquartile ranges (IQR) for continuous variables. All patients at baseline and only untreated patients at M6 were considered for the analyses. Non parametric tests were used to avoid the impact of potential outlier values in a small study. Comparisons between groups were performed using the Mann-Whitney test. The Wilcoxon matched-pairs test was used to estimate the changes in the different variables throughout the follow-up. The Spearman's non parametric correlation was used to estimate the association of two continuous variables of interest. P-values below 0.05 were considered statistically significant.

### Accession numbers

The UniProtKB (http://www.uniprot.org/) accession numbers for the proteins discussed in this paper are IL-17 (Q16552, IL17_HUMAN) ; IL-1RA (P18510, IL1RA_HUMAN) ; Soluble form of CD14 (P08571, CD14_HUMAN) ; MIP-1α (P10147, CCL3_HUMAN) ; I-FABP (P12104, FABPI_HUMAN) ; CCR4 (P51679, CCR4_HUMAN) ; CCR6 (P51684, CCR6_HUMAN) ; CXCR3 (P49682, CXCR3_HUMAN) ; Ki-67 (P46013, KI67_HUMAN) ; CD38 (P28907, CD38_HUMAN) ; CD25 (P01589, IL2RA_HUMAN) ; CD127 (P16871, IL7RA_HUMAN) ; FoxP3 (Q9BZS1, FOXP3_HUMAN).

## Supporting Information

Figure S1 **Flow cytometry dot plots showing the expression of HLA-DR, CD38 and Ki-67 on gated CD3^+^CD8^+^ T cells.** The figure illustrates data obtained from a representative patient at baseline (left panels), and two patients at month 6, including an untreated patient (middle panels) and a patient under antiretroviral therapy (right panels).
(TIF)

Figure S2 **Th17 cell frequency in patients with primary HIV infection.** Th17 cells were assessed following 5 h PMA/ionomycin stimulation of fresh isolated CD4 T cells. The frequency of IL-17-expressing cells was assessed by flow-cytometry (one representative staining is illustrated in panel A). Panel B depicts the results of Th17 frequencies at baseline and month 6. Purple lines indicate patients receiving antiretroviral therapy between baseline and month 6; grey lines indicate untreated patients. Th17 cell frequencies did not differ between baseline and M6 (Wilcoxon rank test) and between treated and untreated patients at M6 (Mann-Whitney test).
(TIF)

Figure S3 **Plasma levels of sCD14 and IL-1RA in patients with primary HIV infection.** Concentrations of sCD14s (Panel A) and IL-1RA (Panel B) were measured in plasma samples of patients at baseline and month 6. sCD14 and IL-1RA plasma levels did not differ between baseline and M6 in untreated and in treated patients (Wilcoxon rank test) and between treated and untreated patients at M6 (Mann-Whitney test).
(TIF)
