# Supplementary figures and images for: The Th17/Treg Ratio, IL-1RA and sCD14 Levels in Primary HIV Infection Predict the T-cell Activation Set Point in the Absence of Systemic Microbial Translocation
Source: PLoS Pathog. 2013 Jun 20;9(6):e1003453. doi: 10.1371/journal.ppat.1003453 (PMC3688532; doi:10.1371/journal.ppat.1003453)

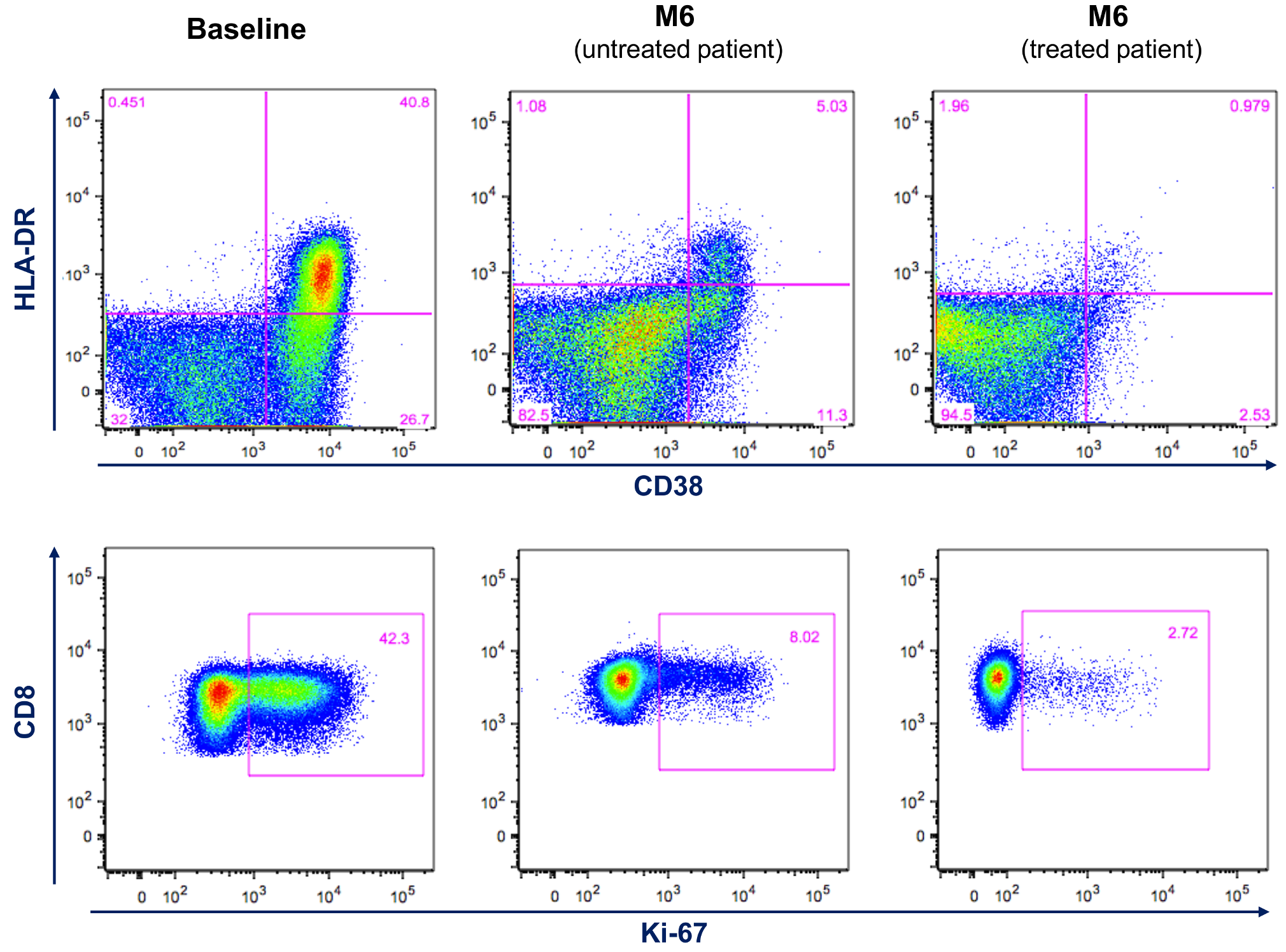

Supplement: Figure S1 — Flow cytometry dot plots showing the expression of HLA-DR, CD38 and Ki-67 on gated CD3+CD8+ T cells. The figure illustrates data obtained from a representative patient at baseline (left panels), and two patients at month 6, including an untreated patient (middle panels) and a patient under antiretroviral therapy (right panels). (TIF) [file ppat.1003453.s001.tif]

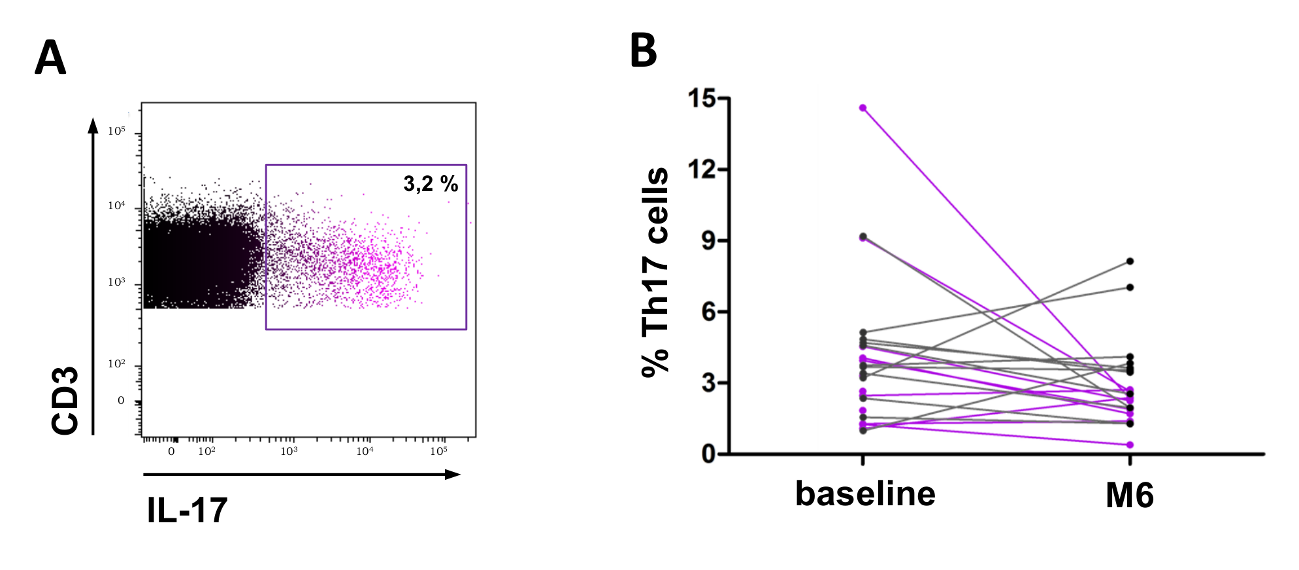

Supplement: Figure S2 — Th17 cell frequency in patients with primary HIV infection. Th17 cells were assessed following 5 h PMA/ionomycin stimulation of fresh isolated CD4 T cells. The frequency of IL-17-expressing cells was assessed by flow-cytometry (one representative staining is illustrated in panel A). Panel B depicts the results of Th17 frequencies at baseline and month 6. Purple lines indicate patients receiving antiretroviral therapy between baseline and month 6; grey lines indicate untreated patients. Th17 cell frequencies did not differ between baseline and M6 (Wilcoxon rank test) and between treated and untreated patients at M6 (Mann-Whitney test). (TIF) [file ppat.1003453.s002.tif]
